# De Novo Design of an Androgen Receptor DNA Binding Domain‐Targeted peptide PROTAC for Prostate Cancer Therapy

**DOI:** 10.1002/advs.202201859

**Published:** 2022-08-15

**Authors:** Bohan Ma, Yizeng Fan, Dize Zhang, Yi Wei, Yanlin Jian, Donghua Liu, Zixi Wang, Yang Gao, Jian Ma, Yule Chen, Shan Xu, Lei Li

**Affiliations:** ^1^ Department of Urology The First Affiliated Hospital Xi'an Jiaotong University #277 Yanta West Road Xi'an China

**Keywords:** androgen receptor splice variant‐7 (AR‐V7), peptide drug, prostate cancer, proteolysis‐targeting chimera (PROTAC)

## Abstract

Androgen receptor splice variant‐7 (AR‐V7), one of the major driving factors, is the most attractive drug target in castration‐resistant prostate cancer (CRPC). Currently, no available drugs efficiently target AR‐V7 in clinical practice. The DNA binding domain (DBD) is indispensable for the transcriptional activity of AR full length and AR splice variants, including AR‐V7. Based on the homodimerization structure of the AR DBD, a novel peptide‐based proteolysis‐targeting chimera (PROTAC) drug is designed to induce AR and AR‐V7 degradation in a DBD and MDM2‐dependent manner, without showing any activity on other hormone receptors. To overcome the short half‐life and poor cell penetrability of peptide PROTAC drugs, an ultrasmall gold (Au)‐peptide complex platform to deliver the AR DBD PROTAC in vivo is developed. The obtained Au‐AR pep‐PROTAC effectively degrades AR and AR‐V7 in prostate cancer cell lines, particularly in CWR22Rv1 cells with DC_50_ values 48.8 and 79.2 nM, respectively. Au‐AR pep‐PROTAC results in suppression of AR levels and induces tumor regression in both enzalutamide sensitive and resistant prostate cancer animal models. Further optimization of the Au‐AR pep‐PROTAC can ultimately lead to a new therapy for AR‐V7‐positive CRPC.

## Graphical abstract

A novel peptide PROTAC targeting androgen receptor DNA binding domain via AI‐Rosetta assisted design for castration‐resistant prostate cancer therapy is presented.

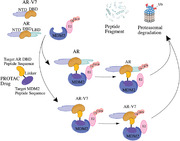

## 1 Introduction

Prostate cancer is the most common and second leading lethal cancer among men.^[^ ^1^ ^]^ The androgen receptor (AR) plays a key role in the development and progression of prostate cancer.^[^ ^2^ ^]^ With the development of second‐generation AR antagonists including abiraterone,^[^ ^3^ ^]^ enzalutamide,^[^ ^4^ ^]^ and darolutamide,^[^ ^5^ ^]^ the treatment and survival of prostate cancer patients have been dramatically improved.^[^ ^6^ ^]^ Although more than 80% of prostate cancer patients initially respond to androgen deprivation therapy (ADT), they eventually develop castration‐resistant prostate cancer (CRPC) after 24–36 months.^[^ ^7^ ^]^ Molecular mechanisms of ADT resistance are largely driven by AR aberrations including AR protein overexpression,^[^ ^8^ ^]^ AR gene amplification,^[^ ^9^ ^]^ AR gene mutations,^[^ ^10^ ^]^ and AR splice variants.^[^ ^11^ ^]^ The expression of AR splice variants, especially AR splice variant‐7 (AR‐V7), is a crucial factor in CRPC progression and drug resistance.^[^ ^12^ ^]^ All second‐generation AR antagonists including enzalutamide and darolutamide target the ligand‐binding domain (LBD) in AR.^[^ ^13^ ^]^ Compared with the full‐length AR structure, AR‐V7 lacks the LBD.^[^ ^14^ ^]^ Functionally, the AR‐V7 could be constitutively active without androgen binding, thereby representing an important means of escape from ADT in prostate cancer.^[^ ^15^ ^]^ Moreover, enzalutamide has been shown to increase the expression of the AR‐V7 variant but not full‐length AR protein level in prostate cancer patients,^[^ ^16^ ^]^ making AR‐V7 a rational and promising target in CRPC.

Some great efforts have been made by scientists to develop novel inhibitors targeting AR‐V7.^[^ ^17^ ^]^ While, only AR‐V7 targeting compound EPI‐506 (NCT02606123)^[^ ^18^ ^]^ and its derivative EPI‐7386 (NCT04421222, NCT05075577)^[^ ^19^ ^]^ targeting AR N‐terminal domain (NTD) have entered into clinical phase 1/2 study for CRPC patients. However, no drugs targeting AR‐V7 could ultimately enter into clinical use indicating there is still an urgent need for developing novel AR‐V7 targeting therapies. Structural studies have shown that homodimerization of AR via their DNA binding domain (DBD) is essential for its transcriptional activity^[^ ^20^ ^]^ and that the DBD is highly conserved across different AR mutants and variants, including AR‐V7 and other AR variants.^[^ ^21^ ^]^ Also, AR DBD forms a dimerization interface distinct from other hormone receptors making the dimerization interface a unique drug target. The homo‐dimeric interaction interface of the AR DBD is a typical protein‐protein interaction interface that adopts a flat, extended conformation; the binding of small molecules is thus energetically difficult, although the binding of peptide drugs is feasible.^[^ ^20^ ^]^ Based on the protein structure, via Artificial Intelligence (AI)‐aided peptide drug design with a hotspot‐centric Rosetta computational approach,^[^ ^22^ ^]^ we designed a novel peptide antagonist with high binding affinity for the AR DBD.

Proteolysis‐targeting chimera (PROTAC) drug design strategy provides a new opportunity for cancer therapeutic development involving the induction of protein degradation.^[^ ^23^ ^]^ A PROTAC drug is a heterobifunctional molecule containing one ligand that binds to the target protein of interest and a second ligand for an E3 ligase.^[^ ^24^ ^]^ As the AR protein plays a key role in CRPC, several small‐molecule PROTAC drugs target the full‐length AR, including ARV‐110,^[^ ^25^ ^]^ ARCC‐4,^[^ ^26^ ^]^ and ARD‐69,^[^ ^27^ ^]^ have achieved great efficacy. However, only one PROTAC drug targeting AR‐V7, MTX–23—has been developed.^[^ ^28^ ^]^ Nevertheless, compared with full‐length AR PROTAC drugs (DC_50_ < 1 nM), MTX‐23 (DC_50_ ≈ 2 µM for AR) is much less effective because of the protein structure of the AR DBD. The published data indicated that there is still an unmet need for an AR‐V7‐targeting PROTAC. Among various inhibitors with therapeutic potential, peptide drugs show higher potency, greater selectivity, and lower toxicity than low molecular weight compounds due to their larger interface with the target protein.^[^ ^29^ ^]^ Therefore, peptide PROTAC drugs have more advantages against targets lacking definite binding pockets such as AR‐V7. Starting from our designed AR DBD‐targeting peptide and a reported peptide sequence targeting the E3 ligase MDM2,^[^ ^30^ ^]^ we developed a novel peptide‐based PROTAC drug that induced the degradation of both AR and AR‐V7 in addition to other variants.

Although PROTAC drugs have become an important modality for certain targets, there are still protein targets that do not have certain small‐molecule binding sites, which is necessary for PROTAC design. In this case, except from small molecular PROTAC drugs other PROTAC forms are beginning to gain attention. Some conventional PROTAC drugs have been designed to target different targets like Bruton's tyrosine kinase,^[^ ^31^ ^]^ EGFR,^[^ ^32^ ^]^ KRAS,^[^ ^33^ ^]^ etc., which all showed great therapeutic potential. Oligo‐based PROTAC (O'PROTAC) have been developed to target DNA‐binding proteins^[^ ^34^ ^]^ and transcription factors.^[^ ^35^ ^]^ These different PROTAC design has greatly enriched the development of the whole research field. Different from small molecules, peptide PROTAC is regarded as bioPROTAC.^[^ ^36^ ^]^ The concept of PROTAC was proved by a peptide PROTAC design targeting IkBalpha. Poor membrane penetration and short half‐life limit the direct use of peptide PROTAC, while new technologies have been developed to overcome the disadvantages to promote peptide PROTAC development. Cyclic peptide PROTAC^[^ ^37^ ^]^ and staple peptide PROTAC^[^ ^38^ ^]^ designs have been proved feasible for peptide PROTAC design. There is still a lack of rational design strategies for peptide PROTAC drugs, although peptide PROTAC has the advantage of better safety and potency. Protein structure prediction and drug screening by artificial intelligence have attracted a lot of attention and greatly improved the efficiency of novel drug discovery.^[^ ^39^ ^]^ Here, we introduced the Rosetta system developed by Dr. Baker's group^[^ ^40^ ^]^ and the protocol developed by Colin A. Smith and Tanja Kortemme^[^ ^22^ ^]^ into peptide PROTAC drug rational design. Separately, we have designed novel targeted peptide sequences for interest protein‐AR DBD and E3 ligase‐MDM2 with Rosetta assisted. Our designed AR DBD targeting peptide PROTAC drug forcibly linked MDM2 to the AR DBD and induced degradation of both AR‐V7 and AR. To overcome the disadvantage of peptide PROTAC – short half‐life and poor cell penetrability,^[^ ^41^ ^]^ we developed a universal one‐step ultrasmall gold (Au)‐peptide PROTAC protocol for delivery of the peptide PROTAC drug in potential clinical applications, inspired by published protocols.^[^ ^42^ ^]^

In summary, we report a peptide PROTAC designed by the AI system Rosetta that targets the AR DBD and is doped with novel ultrasmall Au nanoparticles as a delivery system, and we demonstrate its efficient induction of AR and AR‐V7 degradation both in vitro and in vivo, therefore indicating its potential for CRPC therapy and pharmacological application.

## 2 Results

### 2.1 AR Pep‐PROTAC Drug Design Strategy and Binding Assays

**Figure 1:**
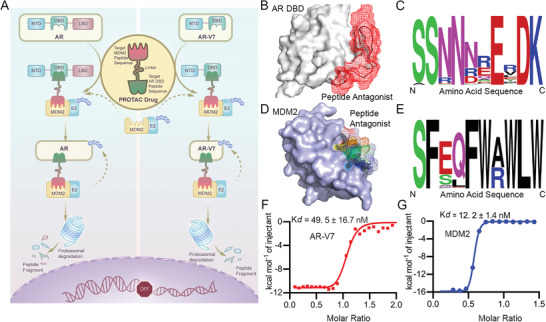
Schematic diagram and in silico design of an AR DBD PROTAC drug. A) Schematic diagram of an AR DBD‐specific PROTAC drug hijacking the E3 ligase MDM2 to degrade full‐length AR as well as AR‐V7. B) The binding structure of the designed peptide ligand (red) and the homodimeric structure of the AR DBD (gray, Protein Data Bank ID: 1r4i). C) Sequence logos of predicted sequence mutations for AR DBD‐targeting peptides. The peptide sequences are arranged in a single letter pattern. N, amino terminus; C, carboxyl terminus. The score for each amino acid was determined using the all‐atom Rosetta scoring function; a larger score means a higher affinity for the AR DBD. D) The structure of the protein complex between a 12‐aa peptide ligand (rainbow) and the MDM2 NTD (purple, Protein Data Bank ID: 3LNZ). E) Sequence logos for predicted sequence mutations for the MDM2‐targeting peptide. The peptide sequences are arranged in a single letter pattern. N, amino terminus; C, carboxyl terminus. The score for each amino acid was determined using the all‐atom Rosetta scoring function; a larger score means a higher affinity for MDM2. F) The binding affinity between the AR pep‐PROTAC drug and AR‐V7 was measured by ITC. The dissociation constant for binding between the AR pep‐PROTAC drug and AR‐V7 was 49.5 nM. G) The binding affinity between the AR pep‐PROTAC drug and MDM2 was measured by ITC. The dissociation constant for binding between the AR pep‐PROTAC drug and MDM2 was 12.2 nM.

A PROTAC is composed of a substrate‐targeting molecule, an E3 ligase‐recruiting element, and a linker. PROTACs can hijack the intracellular ubiquitin‐proteasome system to degrade target proteins. As shown in **Figure** **1**A, our designed PROTAC drug targets the DBD of AR and requires the E3 ligase MDM2. To target the AR DBD, a 16‐amino‐acid (aa) peptide was derived from the homo‐dimeric structure of the AR DBD (Figure 1B) by Dr.Yuval Sedan and colleagues published Rosetta protocol. To achieve a higher‐affinity AR DBD‐targeting peptide drug sequence, virtual amino acid screening was performed at key amino acid sites using Rosetta and published protocol. Rosetta calculates an overall interface binding score and a fold stability score for each amino acid in the given protein‐protein interaction interface. As shown in Figure 1C, sequence logos are shown for AR targeting peptide prediction, the bigger amin acid logo represents a higher affinity with AR DBD. Sequence logos were generated with LOLA (University of Toronto). In addition, Lu et al.^[^ ^30^ ^]^reported a 12‐aa peptide targeting MDM2 termed PMI identified by phage display (Figure 1D), and we also performed virtual amino acid screening to achieve a higher‐affinity MDM2 binding sequence according to the same protocol. By adding a flexible linker sequence (GGSGG) between the AR DBD targeting sequence and MDM2 targeting sequence, we obtained the designed peptide PROTAC sequence: YLCSSNNNRERDKFRRGGSGGTSFEQFWAWLWP.

The designed peptide PROTAC drug was achieved by solid‐phase peptide synthesis, analytical high‐performance liquid chromatography (HPLC), and electrospray ionization mass spectrometry (ESI‐MS) results of the synthesized peptide PROTAC drug are shown in Figure S1, Supporting Information. For the designed novel peptide PROTAC drug sequence, the high affinity between peptide PROTAC and MDM2/AR‐V7 is the most important feature. To determine the binding affinity between the AR pep‐PROTAC drug and AR‐V7/MDM2, isothermal titration calorimetry (ITC) experiments were performed. The MDM2 and AR‐V7 proteins were obtained by overexpression in *E. coli* as published protocol.^[^ ^43^ ^]^ Importantly, the AR pep‐PROTAC drug is bound to MDM2 with a K*d* of 12.2 nM and bound to AR‐V7 with a K*d* of 49.6 nM (Figure 1F,G; Figure S2, Supporting Information). We determined the ability of AR pep‐PROTAC for inhibiting the AR DBD dimer complex with DNA as AR DBD PROTAC targeting the AR DBD dimer interface. The AR DBD domain protein was first overexpressed in *E. coli* as published protocol and labeled with rhodamine B. As shown in Figure S4, Supporting Information, with the increase of AR pep‐PROTAC concentration, the fluorescence polarization (FP) value of rhodamine B labeled AR DBD decreased indicating that AR pep‐PROTAC disturbed AR DBD dimer. The half‐maximal effective concentration (EC_50_) of AR DBD dimer inhibition by AR DBD PROTACr is 1.8 µM, in presence of 200 nM of AR DBD dimer. The maximum FP was taken to indicate 100% dimer of AR DBD, and the minimum was taken to indicate the monomer AR DBD. As the MDM2 recruiting peptide sequence template was from PMI which is a MDM2/MDMX dual‐target peptide, we also performed an ITC experiment between MDMX and AR pep‐PROTAC. As shown in Figure S5, Supporting Information, the K*d* between MDMX and AR pep‐PROTAC is 1.43 µM.

### 2.2 Synthesis and Characterization of Ultrasmall Au Nanoparticles Loaded with the AR Pep‐PROTAC Drug

**Figure 2:**
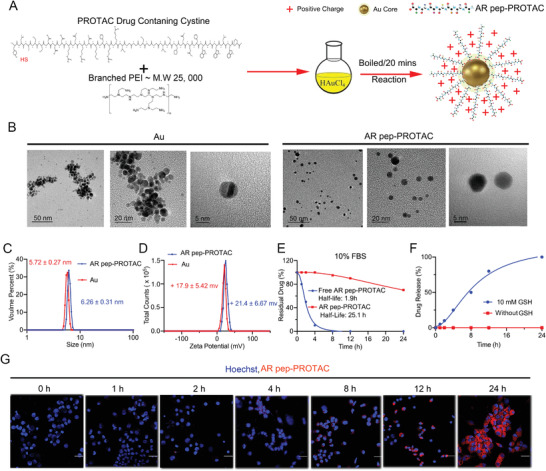
Design and Characterization of the Au‐AR pep‐PROTAC Drug. A) Schematic depiction of the synthesis process of the positive surface Au‐AR pep‐PROTAC drug. HAuCl_4_, chloroauric acid. We developed a stable peptide‐loaded Au nanoparticle system loaded with the AR pep‐PROTAC drug after a 20 min reaction by adding the AR pep‐PROTAC drug‐containing cysteine and branched PEI (MW 25 000 Da) to HAuCl_4_. B) TEM analysis showed that the diameters of the Au nanoparticles and Au‐AR pep‐PROTAC drug were approximately 5.00 nm. C) Hydrodynamic diameter distributions of Au nanoparticles and Au‐AR pep‐PROTAC. The hydrodynamic diameter of the Au nanoparticles was 5.72 nm, and the hydrodynamic diameter of Au‐AR pep‐PROTAC was 6.26 nm. D) The surface zeta potential of the Au nanoparticles and Au‐AR pep‐PROTAC drug was measured in 20 mM Tris‐HCl at pH 7.4. The surface charge of the Au nanoparticles was +17.9 mV, and the surface charge of the Au‐AR pep‐PROTAC was + 21.4 mV. E) The serum resistance of the free AR pep‐PROTAC drug and Au‐AR DBD PROTAC drug was tested in PBS containing 10% serum. The half‐life of the free AR pep‐PROTAC drug was only 1.9 h, while the half‐life of the Au‐AR pep‐PROTAC was 25.1 h. The AR pep‐PROTAC drugs were quantified by HPLC. The “Residual Drug” means the AR pep‐PROTAC still binding to Au nanoparticles without degradation. F) Release of the AR pep‐PROTAC drug from Au‐AR pep‐PROTAC nanoparticles in 10 mM glutathione solution (pH 7.4) to mimic the intracellular redox environment. The AR pep‐PROTAC drug was quantified by HPLC. G) Confocal micrographs of C4‐2 cells incubated with 12.5 nM Au‐AR pep‐PROTAC drug labeled with rhodamine (red). Nuclei are stained with Hoechst 33 342 (blue). All images were acquired with the same excitation wavelength and detector gain settings (scale bar: 50 µm).

To synthesize AR pep‐PROTAC drug‐conjugated ultrasmall Au nanoparticles with high peptide drug‐loading efficiency and stability, we developed a facile, customizable, one‐step chemistry. As shown in **Figure** **2**A, the peptide AR pep‐PROTAC drug, which contains a cysteine in its sequence and branched poly(ethylenimine) (PEI) (MW ≈ 25,000 Da), was mixed with chloroauric acid. After boiling for 20 mins, we achieved the ultrasmall Au nanoparticles loaded with AR pep‐PROTAC drug. Via transmission electron microscopy (TEM) and dynamic light scattering, we evaluated the diameter of the bare Au nanoparticles and AR DBD PROTAC drug‐loaded Au nanoparticles to be approximately 5 nm (Figure 2B,C). The positive surface potential of Au nanoparticles could enable cell penetration (Figure 2D) of Au‐AR pep‐PROTAC nanocomplex.

To examine the serum stability of the Au‐AR pep‐PROTAC, we suspended the Au‐AR DBD PROTAC drug and free AR DBD PROTAC drug separately in PBS containing 10% fetal bovine serum (FBS) and measured time‐dependent changes by reversed‐phase HPLC (RP‐HPLC) (Figure 2E). The results proved that Au nanoparticles could greatly prolong the half‐life of the AR pep‐PROTAC drug. Compared with free AR pep‐PROTAC drug, the half‐life of the AR pep‐PROTAC drug‐loaded Au nanoparticles in serum was increased by 13.2‐fold. As the Au‐AR pep‐PROTAC conjugates to Au nanoparticles through gold thiol bond, which could be reduced under intracellular redox conditions. GSH as a frequently‐used mild reducing agent could reduce the gold‐thiol bond to release AR pep‐PROTAC from Au nanoparticles. Also, GSH is usually used to mimic intracellular redox conditions.^[^ ^44^ ^]^ Au‐AR pep‐PROTAC facilitate controlled drug release in the cellular environment (Figure 2F). Additionally, we examined AR pep‐PROTAC drug internalization using confocal microscopy. The AR pep‐PROTAC drug was labeled with rhodamine, as shown in Figure 2G. Over time, the concentration of the AR DBD PROTAC drug entering the cell gradually increased. To test the stability of Au‐AR pep‐PROTAC in an acidic environment, Au‐AR pep‐PROTAC with 1 mg ml^−1^ concentration was separately incubated in pH 3.0, 5.0, and 7.4 buffer for 24 h. The remanent AR pep‐PROTAC drug was quantified by HPLC. As shown in Figure S6, Supporting Information, only pH3.0 showed a little effect on Au‐AR pep‐PROTAC, Au‐AR pep‐PROTAC remained stable at pH7.4 and 5.0.

By constructing an ultrasmall Au‐AR pep‐PROTAC drug complex, we achieved the goal of highly efficient delivery of the peptide AR DBD PROTAC drug. Compared with larger nanoparticles, ultrasmall nanoparticles have been reported to exhibit more efficient renal clearance in vivo, with almost no accumulation in filter organs and enhanced permeability and retention (EPR) in tumors.^[^ ^45^ ^]^ The ultrasmall Au‐AR DBD PROTAC drug complex allows the potential clinical use of the peptide AR DBD PROTAC drug.

### 2.3 The Au‐AR pep‐PROTAC Drug Induces AR and AR‐V7 Degradation, Inhibits the Proliferation of both AR‐ and AR‐V7‐Positive Prostate Cancer Cells

**Figure 3:**
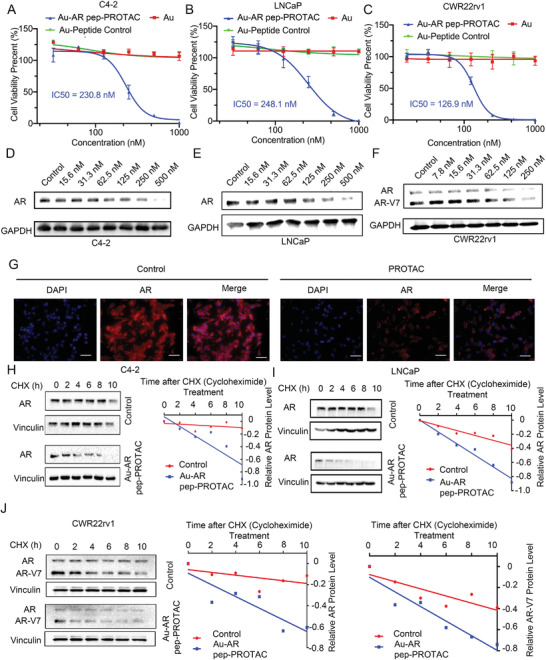
The Au‐AR pep‐PROTAC drug effectively induces the degradation of AR and AR‐V7 in vitro. A) Cell viability assay of C4‐2 cells after 48 h of treatment with varying concentrations of the Au‐ARpep‐PROTAC, Au nanoparticles, and Au‐Peptide Control. The Au nanoparticles and Au‐Peptide Control showed no toxicity in C4‐2 cells, while the Au‐AR pep‐PROTAC showed dose‐dependent growth inhibition in C4‐2 cells, with an IC_50_ = 230.8 nM. B) Cell viability assay of LNCaP cells after 48 h of treatment with varying concentrations of the Au‐AR pep‐PROTAC, Au nanoparticles, and Au‐Peptide Control. The Au nanoparticles and Au‐Peptide Control showed no toxicity in LNCaP cells, while the Au‐AR DBD PROTAC showed dose‐dependent growth inhibition in LNCaP cells, with an IC_50_ = 248.1 nM. C) Cell viability assay of CWR22Rv1 cells after 48 h of treatment with varying concentrations of the Au‐AR pep‐PROTAC, Au nanoparticles, and Au‐Peptide Control. The Au nanoparticles and Au‐Peptide Control showed no toxicity in CWR22Rv1 cells, while the Au‐AR DBD PROTAC showed dose‐dependent growth inhibition in CWR22Rv1 cells, with an IC_50_ = 126.9 nM. D‐F) IB analysis of AR in C4‐2 (D), LNCaP (E), and CWR22Rv1 (F) cells after 24 h of treatment with the Au‐AR pep‐PROTAC drug. G) Immunofluorescence staining of AR (red) in C4‐2 cells after 24 h of Au‐AR pep‐PROTAC drug treatment. DAPI (blue) was used for nuclear staining. Scale bar: 50 µm. H, I) IB analysis of WCLs from C4‐2 cells (H) and LNCaP cells (I) treated with CHX for the indicated times, with or without 250 nM Au‐AR DBD PROTAC drug treatment. The signal intensity of AR normalized to that of Vinculin was quantified. J) IB analysis of AR and AR‐V7 in CWR22Rv1 cells treated with CHX for the indicated times, with or without 250 nM Au‐AR pep‐PROTAC drug treatment. The signal intensities of AR and AR‐V7 normalized to that of Vinculin were quantified separately.

To evaluate the proliferation‐inhibiting activity of the Au‐AR pep‐PROTAC drug in vitro, we treated C4‐2, LNCaP, and CWR22Rv1 cells with the Au‐AR DBD PROTAC drug at concentrations ranging from 10 to 1000 nM. Bare Au nanoparticles and reversal peptide‐loaded Au nanoparticles were used as negative controls. As the reversal peptide shared the same physicochemical property with designed AR pep‐PROTAC with no binding ability with AR or MDM2, reversal peptide‐loaded Au nanoparticles could be used as an appropriate negative control. As shown in **Figure** **3**A–C, the Au‐AR pep‐PROTAC drug showed a dose‐dependent growth inhibitory effect in C4‐2, LNCaP, and CWR22Rv1 cells, while neither bare Au nanoparticles nor the Au‐Peptide control showed any effects on cancer cells. The half‐maximal inhibitory concentration (IC_50_) values of the Au‐AR pep‐PROTAC drug in LNCaP, C4‐2, and CWR22Rv1 cells were 230.8 nM, 248.1 nM, and 126.9 nM, respectively. Under high concentration (over 500 nM), Au‐AR pep PROTAC show totally inhibition of the growth of AR‐positive prostate cancer cells. The total inhibition of AR positive cancer cells caused by Au‐AR pep‐PROTAC is probably because AR is completely degraded at the protein level in a short period of time as some other small molecular AR targeting PROTAC also showed total inhibition of AR positive cancer cells.^[^ ^46^ ^]^ While these data indicate we need to examine more in‐depth the toxicity of Au‐AR pep‐PROTAC in high concentrations. As the toxicity of Au‐AR pep‐PROTAC is mainly because of Au nanoparticles, we examined cell killing ability of Au nanoparticles alone. As shown in Figure S7, Supporting Information, Au nanoparticles alone showed cell toxicity until above concentration of 20 µM.

To investigate the AR cleavage ability of the Au‐AR DBD PROTAC drug, an immunoblot (IB) analysis of AR was performed. The primary antibody (catalog number:sc‐7305) targeting AR we used is raised against amino acids 299–315 of AR, so we could determine both AR and AR‐V7 at the same time. The Au‐DBD PROTAC drug‐induced the degradation of both full‐length AR and AR‐V7 in both dose‐dependent (Figures 3D–F and S8, Supporting Information) and time‐dependent (Figure S9A,B, Supporting Information) manners. The half‐maximal degradation concentration (DC_50_) values of the Au‐AR pep‐PROTAC drug in LNCaP, C4‐2, and CWR22rv1 cells were 175.2 nM, 193.0 nM, and 48.8 nM (AR)/79.2 nM (AR‐V7), respectively. Immunofluorescence staining of AR in C4‐2 cells after 24 h of Au‐AR pep‐PROTAC drug treatment also confirmed the induction of AR degradation by the Au‐AR pep‐PROTAC drug (Figure 3G). By inhibiting the proteasome degradation pathway, the function of the Au‐AR DBD PROTAC drug was blocked (Figure S9A,B, Supporting Information), indicating that degradation by the AR pep‐PROTAC drug is proteasome pathway‐dependent. Consistent with this result, cells treated with the Au‐AR DBD PROTAC drug did not show an obvious reduction in the AR mRNA level (Figure S9C,D, Supporting Information). As AR pep‐PROTAC was designed to target AR DNA binding domain, AR pep‐PROTAC should also induce other AR variants degradation. As shown in Figure S10, we test the potency of Au‐AR pep‐PROTAC on VCaP cells which expressed AR and AR‐V567es. Our results confirmed that Au‐AR pep‐PROTAC could also induce AR and AR‐V567es degradation in VCaP cells. In CRPC, AR proteins are mainly located in the nucleus, confocal and nucleo‐plasmic separation experiments were performed to test if AR pep‐PROTAC could induce AR degradation in the nucleus. As shown in Figures S11 and S12, Supporting Information, AR pep‐PROTAC interacts with AR both in the cytoplasm and nucleus and Au‐AR pep‐PROTAC could induce AR degradation both in the cytoplasm and nucleus.

As AR pep‐PROTAC was designed to target AR DBD, in theory, the interaction between AR pep‐PROTAC and AR would not be regulated by androgens or antiandrogens. To verify, we have tested the degradation efficacy in presence of androgens and antiandrogens. As shown in Figure S13, Supporting Information, DHT didn't show any inhibition of AR pep‐PROTAC. While enzalutamide could enhance the function of AR pep‐PROTAC in C4‐2 and LNCaP cells because enzalutamide could inhibit AR full‐length function and affect AR protein stability.^[^ ^47^ ^]^ Also to test if the efficacy of the PROTAC could be interfered with by AR‐bound DNA sequences, immunoblot analysis of AR after Au‐AR pep‐PROTAC treatment in the presence of AR‐bound DNA. As shown in Figure S14, Supporting Information, AR‐bound DNA didn't show any effect on Au‐AR pep‐PROTAC function.

To test the specificity of AR pep‐PROTAC on different nuclear hormone receptors DNA binding domains, immunology blot analysis on estrogen receptor (ER), progesterone receptor (PR), mineralocorticoid receptor (MR), and glucocorticoid receptor (GR) has been performed. Au‐AR pep‐PROTAC did not show any activity on ER, PR, MR, and GR in MCF‐7 or T47D cells (Figure S15, Supporting Information). Taking ARV‐110 (clinical trial II) as a representative control, we compared its efficacy with this Au‐AR pep‐PROTAC *in cellulo*. Unlike Au‐AR pep‐PROTAC, ARV‐110 only induced full‐length AR degradation but show little effect on AR‐V7 in CWR22rv1 cells (Figure S16, Supporting Information). The Au‐AR pep‐PROTAC drug potently degraded full‐length AR in C4‐2 and LNCaP cells with a DC_50_ of ≈125 nM and degraded AR‐V7 in CWR22rv1 cells with a DC_50_ of ≈62.5 nM. IB analysis showed that in cells treated with cycloheximide (CHX), the Au‐AR DBD PROTAC drug‐induced full‐length AR and AR‐V7 degradation in time‐ and dose‐dependent manners (Figure 3H–J).

Taking the immunoblot analysis data together, we evaluated that Au‐AR DBD PROTAC could effectively induce both AR and AR‐V7 degradation while showing no effect on other nuclear hormone receptors.

### 2.4 The AR DBD PROTAC Drug Induces AR Degradation in an AR DBD‐ and MDM2‐Dependent Manner

**Figure 4:**
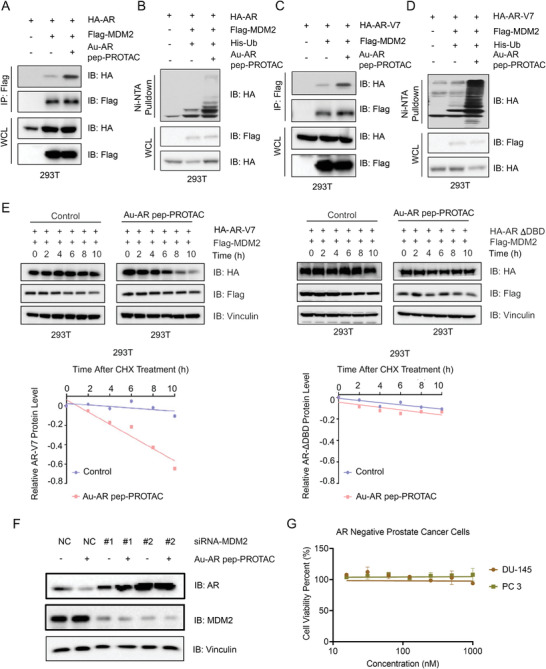
The Au‐AR pep‐PROTAC Drug Degrades AR & AR‐V7 in an AR DBD‐ and MDM2‐Dependent Manner. A) IB analysis of WCLs and anti‐Flag immunoprecipitate (IP) from 293T cells transfected with the indicated plasmids, with or without Au‐AR pep‐PROTAC drug treatment. B) IB analysis of WCLs and Ni–NTA pulldown products derived from 293T cells transfected with the indicated plasmids, with or without Au‐AR pep‐PROTAC drug treatment. Where indicated, 20 µM MG132 was added for 6 h before harvesting the cells. C) IB analysis of WCLs and anti‐Flag immunoprecipitates from 293T cells transfected with the indicated plasmids, with or without Au‐AR pep‐PROTAC drug treatment. D) IB analysis of WCLs and Ni–NTA pulldown products from 293T cells transfected with the indicated plasmids, with or without Au‐AR pep‐PROTAC drug treatment. Where indicated, 20 µM MG132 was added for 6 h before harvesting the cells. E) IB analysis of WCLs from 293T cells transfected with the indicated plasmids and treated with CHX for the indicated times. The signal intensity of AR‐V7 or AR ΔDBD was normalized to that of Vinculin. F) IB analysis of WCLs from C4‐2 cells transfected with siRNAs targeting MDM2 or negative control (NC) siRNAs in the presence of the Au‐AR pep‐PROTAC drug. G) Cell viability assay of AR‐negative DU145 and PC‐3 cells after 48 h of treatment with varying concentrations of the Au‐AR pep‐PROTAC. The data are presented as the mean ± SD values (*n* = 3).

To investigate the cellular drug mechanisms of the AR DBD PROTAC drug, the binding ability between AR and MDM2 in the presence of the AR DBD PROTAC drug in the 293T cell line was investigated. IP (Immunoprecipitation) experiments were performed to detect cellular binding between AR and MDM2. First, 293T cells were transfected with HA‐AR or Flag‐MDM2 plasmids, after 24 h 125 nM Au‐AR DBD PROTAC was added to 293T cells. The cell lysate was collected for IP experiments after 48 h. As shown in **Figure** **4**A, AR is slightly bound to MDM2 under natural conditions. While, in the presence of the drug, the binding ability between AR and MDM2 was greatly enhanced. Consistently, Au‐AR pep‐PROTAC greatly promoted AR protein ubiquitination by E3 ligase‐MDM2 (Figure 4B). Similarity, as shown in Figure 4C&D, Au‐AR pep‐ PROTAC could enhance the binding ability between AR‐V7 and MDM2 and promote AR‐V7 protein ubiquitination. To test if Au‐AR pep‐PROTAC could affect the interaction between endogenous AR and MDM2, endogenous co‐immunoprecipitation experiments were performed. As shown in Figure S17, Supporting Information, Au‐AR pep‐PROTAC could greatly enhance the interaction between endogenous AR and MDM2 in CWR22rv1 cells. Ubiquitination of AR and AR‐V7 were increased only in the presence of the AR pep‐PROTAC drug, indicating that the AR pep‐PROTAC drug induces ubiquitination of AR by hijacking the E3 ligase MDM2 to interact with AR.

To determine the specificity of AR pep‐PROTAC, mutation of amino acids (575‐591) in the AR DBD plasmid was conducted. As shown in Figures S18 and S19, Supporting Information, and Figure 4E, Au‐AR pep‐PROTAC drug failed to induce AR ΔDBD degradation or enhance the binding ability between AR ΔDBD and MDM2.To further confirm the specificity of AR DBD PROTAC, an ITC experiment between AR pep‐PROTAC and AR ΔDBD was performed. The ITC results showed that there was no interaction between AR ΔDBD and AR pep‐PROTAC (Figure S20, Supporting Information). In cells treated with MDM2 siRNA, the AR DBD PROTAC drug could not induce AR degradation (Figure 4F), indicating that the AR DBD PROTAC drug functionally relies on the E3 ligase MDM2. Also, nutlin‐3 was used as MDM2 blocker proved that nutlin‐3 could inhibit function of Au‐AR pep‐PROTAC (Figure S21, Supporting Information). In the AR‐negative cell line DU‐145 and PC3, the Au‐AR pep‐PROTAC drug showed no toxicity on AR negative prostate cancer cells under 1 µM (Figure 4G).

Considering all the in vitro results, we confirmed that AR pep‐PROTAC specific targeting amino acids (575‐591) in AR DBD and action by hijacking MDM2 to interact with AR DBD.

### 2.5 The Au‐AR pep‐PROTAC Drug Potently Inhibits Tumor Growth in C4‐2/CWR22rv1 Xenograft Models In Vivo

To evaluate the therapeutic efficacy of the Au‐AR pep‐PROTAC drug in vivo, we established two subcutaneous xenograft mouse models of prostate cancer with C4‐2 cells (5 × 10^6^, AR‐positive) and CWR22rv1 cells (5 × 10^6^, AR&AR‐V7‐positive). In each of these models, 25 tumor‐bearing mice were randomly divided into 5 groups and treated every three days for three weeks with PBS, bare Au nanoparticles, the Au‐AR pep‐PROTAC drug, Au‐Peptide Control, or enzalutamide at the same dose (2 mg kg^−1^). Two administration routes were tested in the xenograft models, with subcutaneous injection (SC.) in C4‐2 models and intraperitoneal injection (IP.) in CWR‐22rv1 models. All the drug including Au‐AR pep‐PROTAC drug, Au‐Peptide Control and enzalutamide were dissolved in 0.4 mg ml^−1^ in PBS (pH 7.4). Each injection volume was 100 µl and PBS was used as control.

**Figure 5:**
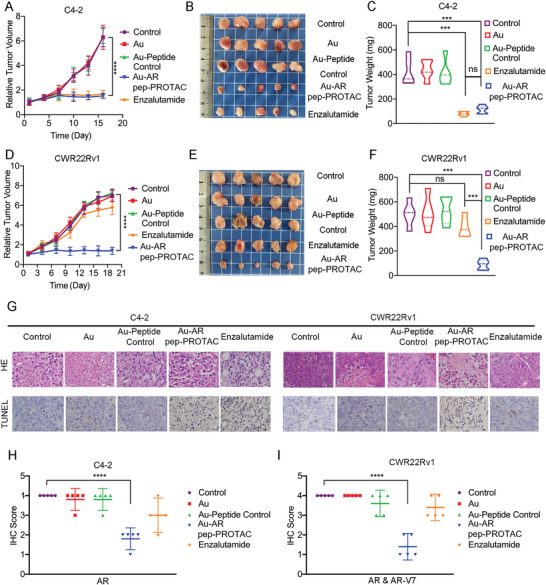
The Au‐AR pep‐PROTAC Drug Inhibits Prostate Tumor Growth In Vivo. A) Tumor growth curves of C4‐2 xenografts in nude mice treated as indicated (*n* = 5 per group). Statistical analysis was performed using the nonparametric Kruskal‐Wallis test; ****, *p* < 0.0001. The data are presented as the mean ± SD values (*n* = 5). B) Photos of C4‐2 tumors excised at the end of the experiment after different drug treatments. C) Average weight of tumors excised from each group of mice at the end of drug treatment. The data are presented as the mean ± SD values (*n* = 5). Statistical analysis was performed using the nonparametric Kruskal‐Wallis test; ***, *p* < 0.001; ns, not statistically significant. The data are presented as the mean ± SD values. D) Tumor growth curves of CWR22Rv1 xenografts in nude mice (*n* = 5 per group). Statistical analysis was performed using the nonparametric Kruskal‐Wallis test; ****, *p* < 0.0001. The data are presented as the mean ± SD values. E) Photos of CWR22Rv1 tumors excised at the end of the experiment after different drug treatments. F) Average weight of tumors excised from each group of mice at the end of drug treatment (*n* = 5). Statistical analysis was performed using the nonparametric Kruskal‐Wallis test; ***, *p* < 0.001; ns, not statistically significant. G) Histopathological analysis of the excised tumors using H&E staining and a TUNEL assay (scale bar: 10 µm). H) IHC analysis of AR in C4‐2 xenografts from each treatment group. IHC scores were determined with ImageJ: 4, highly positive; 3, positive; 2, minimally positive; 1, negative. I) IHC analysis of AR and AR‐V7 CWR22Rv1 xenografts. IHC scores were determined with ImageJ: 4, highly positive; 3, positive; 2, minimally positive; 1, negative. Statistical analysis was performed using the *t*‐test; ****, *p* < 0.0001.

As shown in Figure 4A–F, neither the bare Au nanoparticles nor the Au‐Peptide Control had a therapeutic effect on tumor growth, the Au‐AR DBD PROTAC showed great efficiency in both the C4‐2 and CWR22Rv1 xenograft models, and enzalutamide inhibited tumor growth in the C4‐2 but not the CWR22Rv1 xenograft model. Consistent with the above findings from the in vivo efficacy study, histopathological analysis using hematoxylin and eosin (H&E) staining and terminal deoxynucleotidyl transferase dUTP nick end labeling (TUNEL) revealed a massive amount of apoptotic tumor cells in tissues from Au‐AR DBD PROTAC‐treated mice and enzalutamide‐treated mice (**Figure** **5**G). Immunohistochemical (IHC) analysis of AR & AR‐V7 in each treatment group in the CWR22Rv1 and C4‐2 xenograft models proved that the Au‐AR DBD PROTAC drug effectively induced AR and AR‐V7 degradation in vivo. IHC score analysis was performed by the Image J IHC profiler. Also as shown in Figure S22, Supporting Information, the depletion of AR proteins and apoptosis is relevant in tumor tissue after Au‐AR pep‐PROTAC treatment, the *R*‐value is 0.86.

Compared with AR targeting PROTAC ARV‐110, the animal dosage was 1 mg kg^−1^ or 3 mg kg^−1^ similar amount to Au‐AR pep‐PROTAC (2 mg kg^−1^).^[^ ^48^ ^]^ While for ARD‐61,^[^ ^49^ ^]^ a higher dosage was used in mouse models than Au‐AR pep‐PROTAC.

### 2.6 The PK (pharmacokinetics) and biosafety analysis of Au‐AR pep‐PROTAC

**Figure 6:**
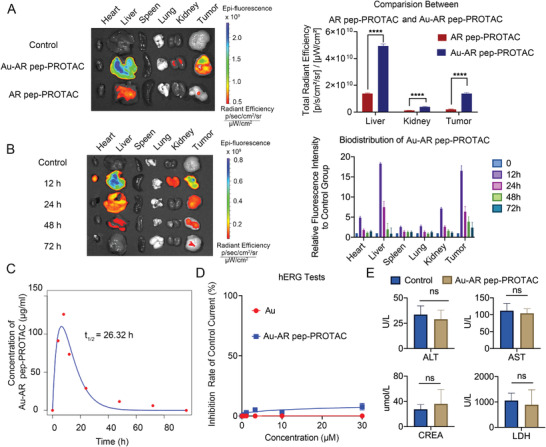
The PK (pharmacokinetics) and biosafety analysis of Au‐AR pep‐PROTAC. A) Comparison of biodistribution between free AR pep‐PROTAC and Au‐AR pep‐PROTAC in CWR22RV1 xenograft models. B) Biodistribution of Au‐AR pep‐PROTAC at different time points in CWR22RV1 xenograft models. C) Pharmacokinetics analysis of Au‐AR pep‐PROTAC by ICP‐MS. D) The hERG tests of Au and Au‐AR pep‐PROTAC on cardiac muscle cells. E) Biosafety evaluation of Au‐AR pep‐PROTAC after 21‐ day treatment of Au‐AR pep‐PROTAC.

To evaluate the pharmacokinetic (PK) properties of Au‐AR pep‐PROTAC drug, AR pep‐PROTAC peptide was first labeled with rhodamine B. We have conducted in vivo mouse pharmacokinetics assay for the peptide PROTAC drug with/without Au with intraperitoneal injection. As shown in **Figure** **6**A, the Au nanoparticle could efficiently accumulate in the tumor site and prolong the half‐life of the AR pep‐PROTAC peptide. As shown in Figure 6B, Au‐AR pep‐PROTAC accumulated within the tumor obviously and could be eliminated in organs after 72 h. To further evaluate the druggability of Au‐AR pep‐PROTAC, PK analysis of Au‐AR DBD PROTAC was performed by ICP‐MS. As depicted in Figures 6C and S23, Supporting Information, the t_1/2_ of Au‐AR DBD PROTAC is 26.3 h. While as shown in publications, the half‐life of majority free peptides is <5 h.

To analyze the toxicity of Au‐AR pep‐PROTAC, hERG tests on cardiac muscle cells up to 30 µM of Au‐AR pep‐PROTAC were performed which is 120 times more than IC_50_ of Au‐AR pep‐PROTAC. As shown in Figure 6D, no cardiac toxicity was observed up to 30 µM for Au‐AR pep‐PROTAC in hERG toxicity assays. Additionally, the safety profile of Au‐AR pep‐PROTAC was confirmed with in vivo model. Au‐AR pep‐PROTAC showed no effect on mouse body weight during drug treatment (Figure S24A, Supporting Information). After 21‐day treatment, we examined the hematological toxicity of Au‐AR pep‐ PROTAC by a routine blood test (Figure S24B, Supporting Information). Biochemical analysis of serum after 21‐day treatment of Au‐AR pe‐PROTAC was performed. The Alanine aminotransferase (ALT), Aspartate aminotransferase (AST), creatinine (CREA), lactate dehydrogenase (LDH) analysis results, and H&E staining of organs showcased no toxicity in the heart, liver, spleen, and kidney (Figure 6E; Figure S24C, Supporting Information). To determine off‐target toxicity, PC3 xenograft animal model was used as negative control. As shown in Figure S25, Supporting Information, Au‐AR pep‐PROTAC didn't show any activity on the PC3 xenograft mice model.

To further detection of toxicity about Au‐AR pep‐PROTAC on liver tissue, we tested toxicity of Au‐AR pep‐PROTAC on AML 12 cells which is a normal mice liver cell line. As shown in Figure S26A, Supporting Information, the IC_50_ of Au‐AR pep‐PROTAC on AML 12 cells is 2.41 µM which is almost ten‐fold compared to IC_50_ in prostate cancer cells. Apoptosis assays also confirmed with the results of cell viability tests (Figure S26B, Supporting Information). ELISA detection of *γ*‐GT(*γ*‐Glutamyl Transferase), ALP(alkaline phosphatase), and AST(Aspartate aminotransferase) in AML 12 cell lysate after 24 h treatment of Au‐AR pep‐PROTAC were also performed (Figure S26C, Supporting Information). To evaluate in vivo liver toxicity of Au‐AR pep‐PROTAC, ELISA detection of *γ*‐GT(*γ*‐Glutamyl Transferase), ALP(alkaline phosphatase), AST(Aspartate aminotransferase), ALT(Alanine aminotransferase), and TBA(total bile acid) in mice serum after 24 h injection of Au‐AR pep‐PROTAC were performed. As shown in Figure S26D, Supporting Information, Au‐AR pep‐PROTAC will affect liver function up to 10 mg kg^−1^.

## 3 Discussion

AR‐V7 is the most attractive target in CRPC,^[^ ^50^ ^]^ while most available drugs targeting AR bind to the LBD, as it contains a ligand‐binding pocket formed by 12 folded helices making AR‐V7 escape from drug targeting.^[^ ^51^ ^]^ However, there is a bottleneck in traditional small molecule drug discovery and development toward AR‐V7.^[^ ^52^ ^]^ Innovatively, we designed a peptide PROTAC drug targeting the AR DBD via AI‐assisted rational design. Via hydrogen bond and charge interactions, peptide drugs can achieve higher binding affinity and specificity than small molecule drugs for targeting the protein‐protein interaction interface.^[^ ^53^ ^]^

In all aspects of drug discovery, the PROTAC drug design strategy is a more promising approach than the traditional antagonist design.^[^ ^54^ ^]^ PROTAC drug design takes advantage of the intrinsic protein degradation mechanism of the ubiquitin‐protease system to eliminate specifically targeted proteins.^[^ ^55^ ^]^ To design our PROTAC, we selected a homodimeric interface, which is essential for AR activity. In addition, MDM2 was selected as the PROTAC‐targeting E3 ligase since it has been reported to be involved in AR ubiquitination and degradation under physiological conditions,^[^ ^37^ ^]^ which increases the degradation efficiency of the designed PROTAC molecule. The DC_50_ value of our designed Au‐AR DBD PROTAC was 125 nM for full‐length AR—16‐fold more effective than MTX‐23—and only 62.5 nM for AR‐V7—5‐fold more effective than MTX‐23.^[^ ^38^ ^]^ These data indicate that peptide PROTAC drugs are more efficient than small‐molecule PROTAC drugs for undruggable targets such as AR‐V7. Peptide PROTAC as an important branch of the field of PROTAC drugs has attracted more and more attention. We firstly report how to induce AI system Rosetta as a common strategy for peptide PROTAC drug design.

Also, we applied nanotechnology for peptide PROTAC delivery. Ultrasmall Au nanoparticles (less than 10 nm in diameter) are promising for biomedical applications because of their relatively low systemic toxicity, rapid kidney clearance, and high tumor accumulation.^[^ ^56^ ^]^ Due to the complexity of peptide molecule side chains, Au nanoparticle‐loaded peptides are usually greater than 50 nm in diameter.^[^ ^57^ ^]^ We developed a fast, stable ultrasmall Au‐peptide system that could avoid the side effects of Au nanoparticles as their ultrasmall size effect. Moreover, this ultrasmall Au‐peptide system could also be applied for universal peptide PROTAC drug delivery.

In summary, we designed a novel peptide‐based PROTAC drug directly targeting the DBD of AR and could induce both AR and AR‐V7 degradation with potential clinical use and provided a universal strategy for peptide PROTAC drug design and delivery.

## 4 Experimental Section

### Peptide Drug Design

Peptide drug design was used Rosetta and “sequence tolerance” protocol. In brief, 10 interface sites was defined at which mutations to all 20 naturally occurring amino acids (except cysteine) will be allowed in the simulations. In a first step, Rosetta calculates an overall interface binding score and a fold stability score for each protein chain in the given protein‐protein complex. Next, Rosetta models and scores nonnative sequences at the interface sites selected for the design. Nonnative sequences at the protein‐protein interface were selected and propagated by a genetic algorithm; for each sequence, the rotameric conformations of each sequence at the interface are optimized by a Monte Carlo simulated annealing protocol and the resulting structural models were scored using the all‐atom Rosetta scoring function. Throughout the simulation, nonnative sequences with folding and binding scores near or significantly better than the starting sequence were saved and considered to be “tolerated”. At the end of a simulation, the frequency with which each amino acid type appeared in the list of tolerated sequences was calculated and used to generate a position‐specific “tolerance profile” for each interface site.

### Peptide Synthesis

Boc amino acids were obtained from Peptides Institute; p‐methyl‐BHA (MBHA) resin was purchased from Applied Biosystems; Tris‐(2‐carboxyethyl) phosphine (TCEP), glutathione (GSH), dichloromethane (DCM), N, N‐dimethylformamide (DMF), acetonitrile, triisopropylsilane (TIPS), N, N‐diisopropylethylamine (DIEA), p‐cresol and ultrapure guanidine hydrochloride (GuHCl) were obtained from Sigma‐Aldrich; and trifluoroacetic acid (TFA) was purchased from Halocarbon.

### ITC (Isothermal Titration Calorimetry) Experiments

ITC (isothermal titration calorimetry) was an established technique that can determine the binding ability between protein and ligands. The signal measured was the heat released upon the interaction of the two reactants. All proteins – MDM2, AR‐V7, or AR ΔDBD were obtained with *E.Coli* expression and purified by affinity chromatography. All peptides were synthesized by solid‐phase peptide synthesis and purified by preparative high‐performance liquid chromatography. For ITC experiments, clean sample cells and injection syringe were cleaned according to the manufacturer's protocol before loading protein and peptide. Rinse the sample cell several times with 2 ml of buffer. Load the sample cell with 0.2 ml of protein solution (all proteins concentration were around 20 µM), being careful to avoid bubble formation, load the syringe with 0.04 ml peptide solution (all peptides concentrations were around 200 µM). Choose the temperature at 25 °C for the ITC experiments. a small volume (0.4 µl) for the first injection was used and keep the subsequent injections at the desired volume (2 µl for 19 injections). Once the experiment had finished, the ITC data was fitted with the analysis software for the instrument.

### Au‐Peptide Complex System Synthesis

To achieve the ultrasmall gold (Au)‐peptide PROTAC complex nanoparticle system, 2 mg peptide was first dissolved in 10 ml water and boiled. After boiling for 3 min, 100 µl 1% HAuCl4 was added to the peptide solution. The molar ratio between Au and AR pep‐PROTAC is 6:1. Then 200 µl 1% branched PEI (25 KD) was added to the solution, and the ultrasmall gold (Au)‐peptide PROTAC complex nanoparticle after boiling for 20 min was achieved. The quantitation of loading peptide was performed by analytic HPLC.

### Cell Culture

293T cells were maintained in Dulbecco's modified Eagle medium containing 10% FBS. LNCap, C4‐2, CWR22Rv1, and DU145 cells were cultured in RPMI 1640 medium containing 10% FBS. VCaP cells were cultured in DMEM medium containing 10% FBS.

### Plasmids

HA‐AR, HA‐AR‐V7, HA‐AR ΔDBD, Flag‐MDM2, and his‐Ub were purchased from Addgene. The plasmids pET‐28a (MDM2), pET‐28a (AR – V7), and pET‐28a (AR ΔDBD) were purchased from GenScript.

### Antibodies

The anti‐AR (sc7305) and anti‐GAPDH (sc‐47724) antibodies were purchased from Santa Cruz. The anti‐AR V567es (ab200827) antibody was purchased from abcam. The anti‐vinculin antibody (V‐4505), anti‐Flag agarose beads (A‐2220), anti‐HA agarose beads (A‐2095), secondary anti‐mouse antibody, and secondary anti‐rabbit antibody were purchased from Sigma‐Aldrich.

### Protein Expression and Purification

*E. coli* BL21 (DE3) cells transformed with pET28‐AR‐V7 expression plasmids were grown to an OD600 of 0.6 at 37°C in LB medium supplemented with 100 µg ml^−1^ Kanamycin. After induction with 1 mM IPTG, cultures were incubated for overnight in 18° before harvesting. The frozen bacteria pellets were resuspended in lysis buffer (50 mM Tris (pH 8.0) and 300 mM NaCl) and lysed by sonication at 4 °C. As AR‐V7 was expressed in the inclusion body, the precipitate was dissolved with 8 M urea and the supernatant was discarded.

Filtered redissolved precipitate was loaded onto a Ni‐NTA column (GE Healthcare) pre‐equilibrated with 8 M urea loading buffer and washed with 5% elution buffer (25 mM Tris pH 8.0, 8 M urea, 500 mM imidazole) followed by a 5%–100% gradient elution with an ÄKTA purifier system. The fractions containing the eluted proteins were concentrated and loaded on Superdex75 16/60 (GE Healthcare) size exclusion chromatography column in 25 mM Tris pH 8.0, 8 M urea. The main protein fractions were dialysis and freeze‐drying for further usage.

For AR‐V7 refolding, AR‐V7 protein powder was dissolved in 6 M GuHCl at 0.2 mg mL^−1^, followed by a six‐fold dilution with Tris‐Hcl containing 0.1 mM TCEP, pH 7.4, and an overnight dialysis against the Tris‐Hcl. After dialysis, protein precipitation was removed by centrifugation. The concentration of AR‐V7 protein was quantified by UV 280 absorption.

### CCK‐8 Cell Viability Assay

Cell viability was evaluated by a cell counting kit‐8 (CCK‐8) assay. Cells were plated at a density of 4 ×10^3^ cells per well in 96‐well plates, and three independent samples were seeded in duplicate for each concentration. Cell viability tests were performed 24 h after treatment with Au, Au‐peptide control, or Au‐AR DBD PROTAC. After a 2 h incubation with CCK‐8 reagent, absorbance values at 450 nm were determined using a microplate reader. The CCK‐8 kit was purchased from Dojindo Laboratories.

### Immunoblotting

Immunoblotting was performed to determine the AR amount after Au‐AR DBD PROTAC treatment. Cells were lysed in EBC buffer (50 mM Tris (pH 7.5), 120 mM NaCl, and 0.5% NP‐40) supplemented with protease inhibitors. Cell lysates were quantified by a Pierce bicinchoninic acid (BCA) Protein Assay Kit (Thermo Fisher). Equal amounts of total proteins were loaded for western blotting analysis. The proteins were separated by SDS‐PAGE gels. Then proteins were transferred to polyvinylidene fluoride (PVDF) membranes and incubated with specific primary antibodies at 4 °C overnight. After incubating with HRP‐conjugated secondary antibody for 1 h at room temperature, the protein immunoreactive signals were tested.

### Immunoprecipitation Assays

For immunoprecipitation (IP) assays, a total of 3 mg of cell lysate was incubated with anti‐flag, anti‐HA, or Ni‐NTA beads for 4 h at 4°C in the absence or presence of AR DBD PROTAC. Subsequently, cell lysates were washed with EBC buffer and the proteins were extracted from the beads by boiling at 95 °C for 5 min. For immunoprecipitation assays, the proteins were separated by SDS‐PAGE gels. Then they have transferred to polyvinylidene fluoride (PVDF) membranes and incubated with specific primary antibodies at 4 °C overnight. After incubating with HRP‐conjugated secondary antibody for 1 h at room temperature, the protein immunoreactive signals were tested.

### Evaluation of Antitumor Activity in C4‐2 and CWR22Rv1 Xenograft Models

Treatment of five groups of mice (5 mice per group) was initiated (i.e., day 1) after tumors were established in 2 weeks as palpable masses (50 mm^3^). Mice in the five medication treatment groups were subjected to a 3‐week treatment regimen with an injection every other day. The tumor length and width were measured with calipers, and the tumor volume was calculated using the following equation: tumor volume (*V*) = length × width^2^/2. Treatment of five groups of mice (five mice per group) was initiated (i.e., day 1) after the tumor had been established after 3 weeks as a palpable mass (around 50 mm^3^ in size). All IHC staining images were scored using ImageJ profiler as published protocol.

### Ethical approval

All experimental procedures involving animals were conducted by institutional guidelines and were approved by the Laboratory Animal Center and Biomedical Ethics Committee of Xi'an Jiaotong University (approval number: 2020(G‐135)).

### Statistics

All data are expressed as the mean ± SD and are representative of triplicate samples, experimental group data were normalized to vehicle treated. Statistics were generated using GraphPad Prism software. Differences among multiple groups and the effects of treatment for data obtained from in vivo and in vitro studies were analyzed by nonparametric Kruskal‐Wallis test for multiple comparisons and *t*‐test for two group comparison. *P*‐values of 0.05 or less were considered statistically significant for single comparisons.

## Conflict of Interest

The authors declare no conflict of interest.

## Supporting information

Supporting Information

## Data Availability

The data that support the findings of this study are available from the corresponding author upon reasonable request.
