# Supplementary material for: De Novo Design of an Androgen Receptor DNA Binding Domain‐Targeted peptide PROTAC for Prostate Cancer Therapy
Source: Adv Sci (Weinh). 2022 Aug 15;9(28):2201859. doi: 10.1002/advs.202201859 (PMC9534960; doi:10.1002/advs.202201859)
Supplement: Supplementary file 1 — Supporting Information [file ADVS-9-2201859-s001.pdf]

De novo Design of an Androgen Receptor DNA Binding Domain-Targeted peptide PROTAC for Prostate Cancer Therapy

Bohan Ma[1,#], Yizeng Fan[1,#], Dize Zhang[1], Yi Wei[1], Yanlin Jian[1],Donghua Liu[1], Zixi Wang[1], Yang Gao[1], Jian Ma[1], Yule Chen[1],Shan Xu[1], Lei Li[1,*]

[1] Department of Urology, The First Affiliated Hospital, Xi’an Jiaotong University, #277 Yanta West Road, Xi’an, China

[#] Bohan Ma and Yizeng Fan contributed equally to this work

*Corresponding author: Lei Li, #277 Yanta West Road, Shaanxi, China, 710061, lilydr@163.com

Conflict of interest: The authors have declared that no conflict of interest exists.

Supplementary Tables and Figures

Figure S1

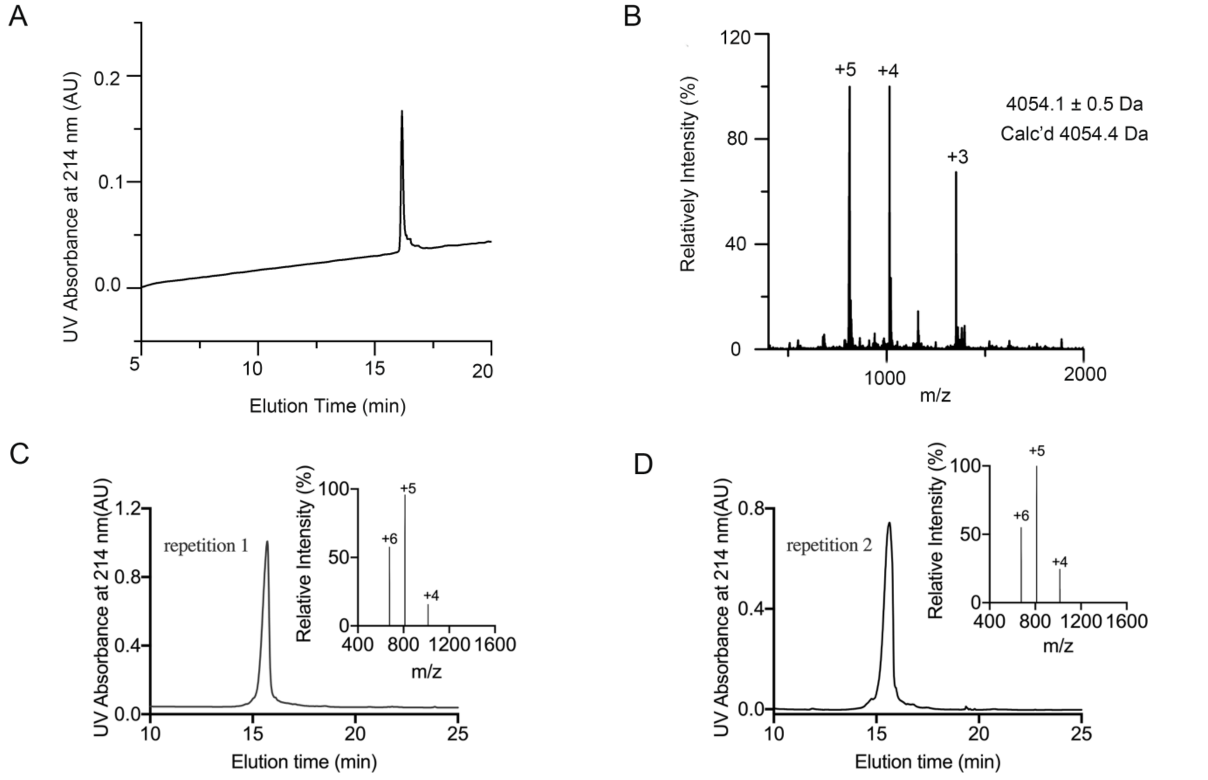

Figure S1. Purification and characterization of AR pep-PROTAC. (A) HPLC characterization of AR pep-PROTAC peptide drug. **(B)** ESI-MS characterization of AR pep-PROTAC peptide drug. **(C)** and **(D)** HPLC and MS characterization of AR pep-PROTAC peptide drug from different batches.

Figure S2

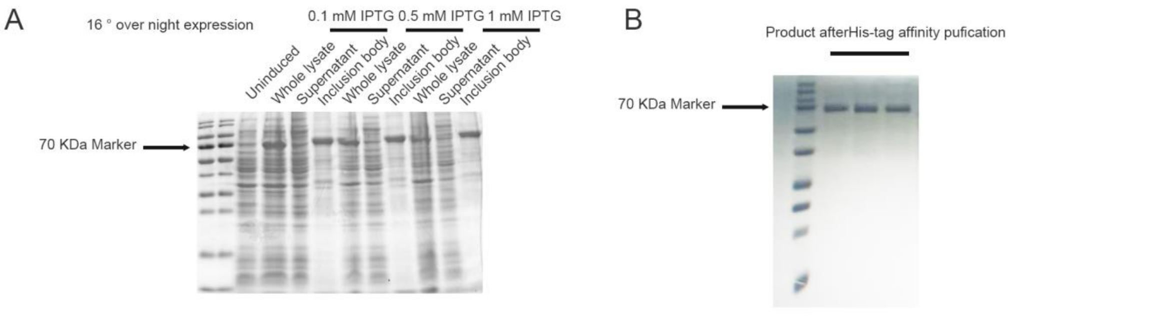

Figure S2. Expression and purification of AR-V7 protein. (A) SDS PAGE assay for the intermediate process of AR-V7 protein expression. **(B)** SDS PAGE assay for purified AR-V7 protein. The protein marker was purchased from Thermo (Cat. No. 26616).

Figure S3

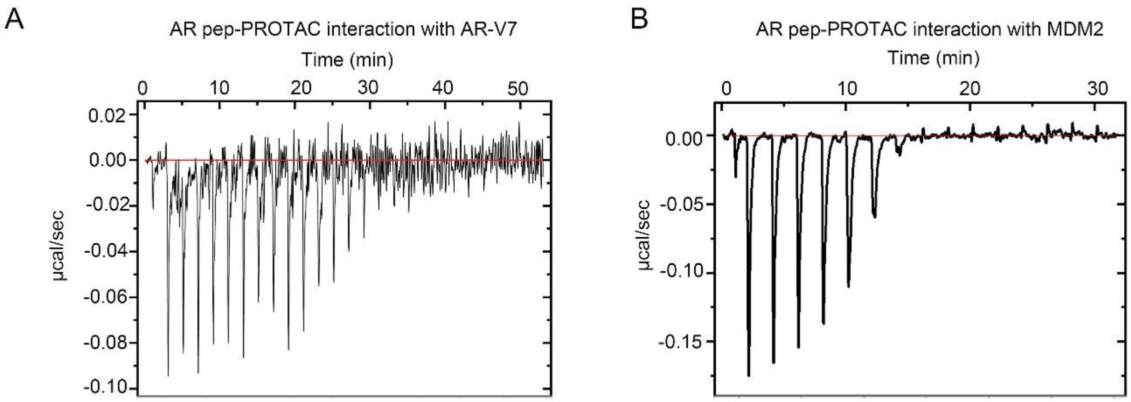

Figure S3. Original ITC data of binding affinity assays. (A) Original ITC data of binding affinity between AR pep-PROTAC with AR-V7. **(B)** Original ITC data of binding affinity between AR pep-PROTAC with MDM2.

Figure S4

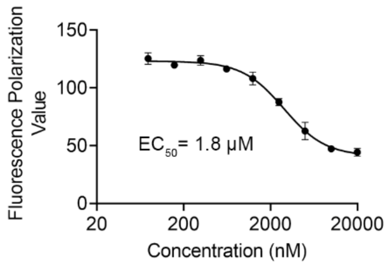

Figure S4. AR pep-PROTAC inhibits AR DBD Dimer/DNA complex formation. The inhibition ability of AR pep-PROTAC for AR DBD dimer complex detection by fluorescence polarization (AR DBD protein was labeled with rhodamine).

Figure S5

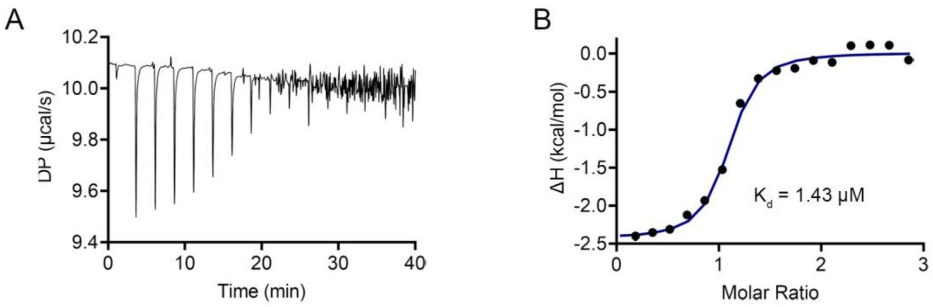

Figure S5. Binding affinity between AR pep-PROTAC with MDMX measured by ITC.

Figure S6

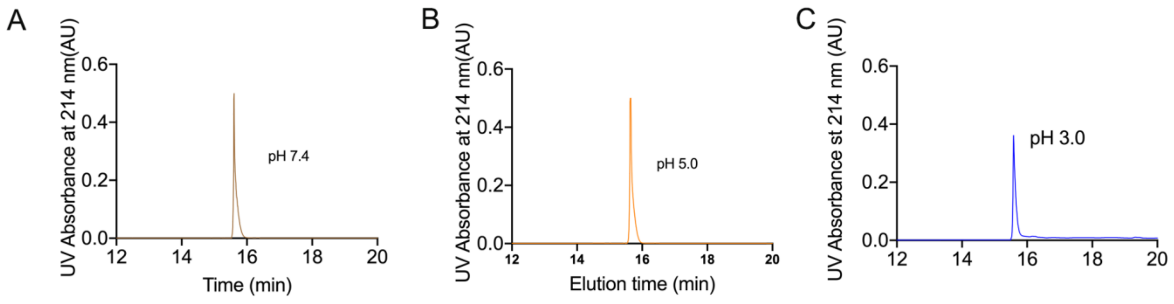

Figure S6. Stability detection of AR pep-PROTAC in an acidic environment.

Figure S7

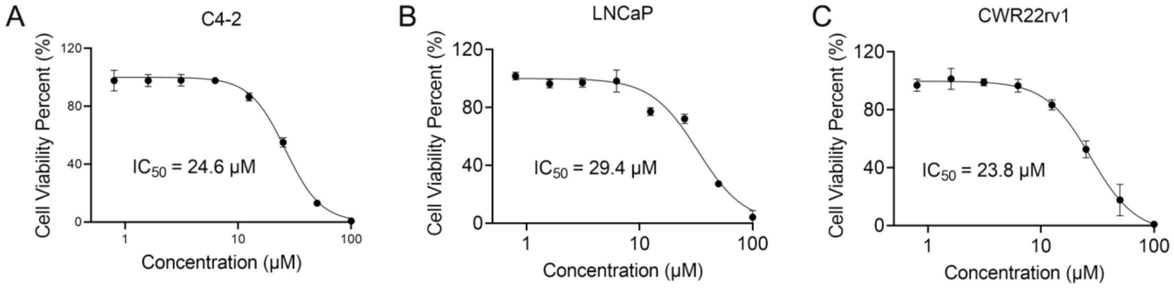

Figure S7. Cell viability assay of C4-2(A), LNCaP (B), and CWR22rv1 (C) with varying concentrations of bare Au nanoparticles.

Figure S8

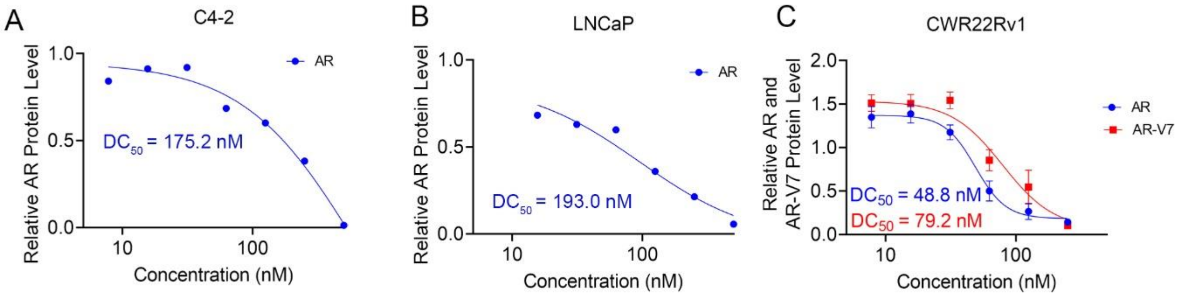

Figure S8. Degradation curve of AR and AR-V7 in C4-2 (A), LNCaP (B), and CWR22rv1 (C) induced by AR pep-PROTAC.

Figure S9

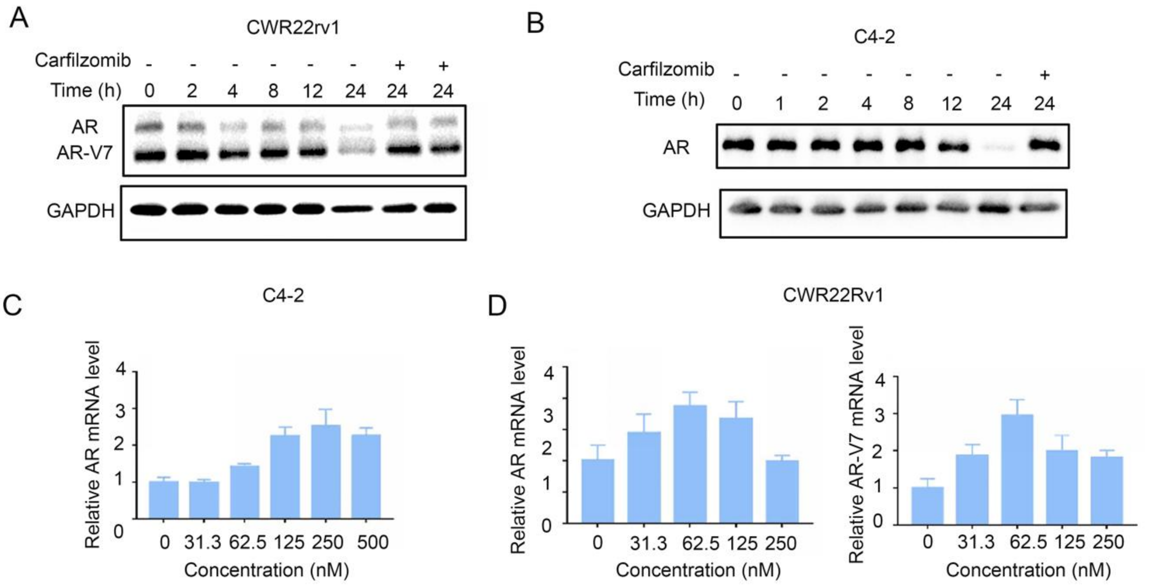

Figure S9. AR pep-PROTAC induces AR degradation in a time- and dose-dependent way but does not reduce AR mRNA level. (A) Au-AR DBD PROTAC induces AR and AR-V7 degradation in a time- and the dose-dependent way in CWR22Rv1 cells and carfilzomib (a proteasome inhibitor) could inhibit Au-AR DBD PROTAC function. **(B)** Au-AR pep-PROTAC induces AR degradation in a time- and the dose-dependent way in C4-2 cells and carfilzomib (a proteasome inhibitor) could inhibit Au-AR pep-PROTAC function. **(C)** Au-AR pep-PROTAC does not reduce the mRNA level of AR in C4-2 cells. **(D)** Au-AR pep-PROTAC does not reduce the mRNA level of AR or AR-V7 in CWR22rv1 cells.

Figure S10

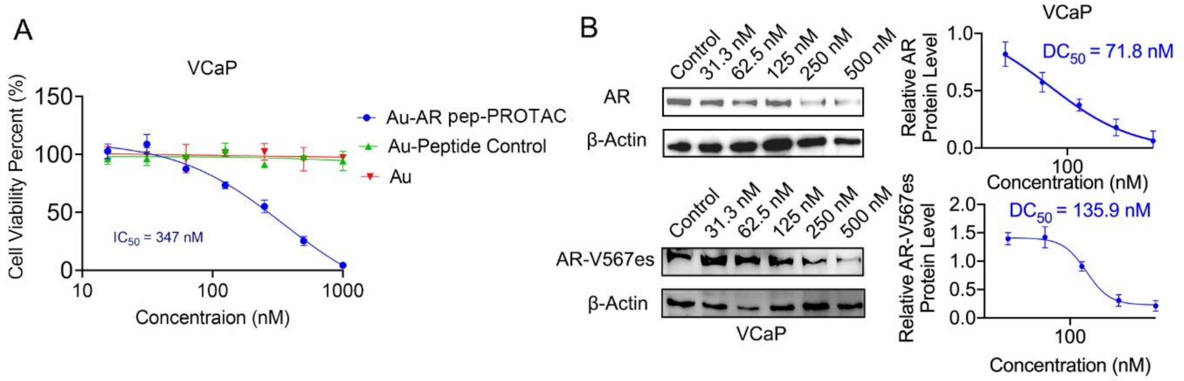

Figure S10. AR pep-PROTAC induces AR and AR-V567es degradation and inhibits VCaP cells growth. (A) Cell viability assay of VCaP cells after 48 h of

treatment with varying concentrations of the AR pep-PROTAC, Au nanoparticles, and Au-Peptide Control. The Au nanoparticles and Au-Peptide Control showed no toxicity in C4-2 cells, while the Au-AR DBD PROTAC showed dose-dependent growth inhibition in C4-2 cells, with an IC50 = 347 nM. **(B)** IB analysis of AR and AR-V567es in VCaP cells after 24 h of treatment with the AR pep-PROTAC drug. This ARv567es antibody (Ab200827, clone EPR15657) is a rabbit monoclonal antibody against human androgen receptor variant 5,6,7es (amino acids 700 to the C-terminus). This antibody does not react with AR-FL and AR-V7.

Figure S11

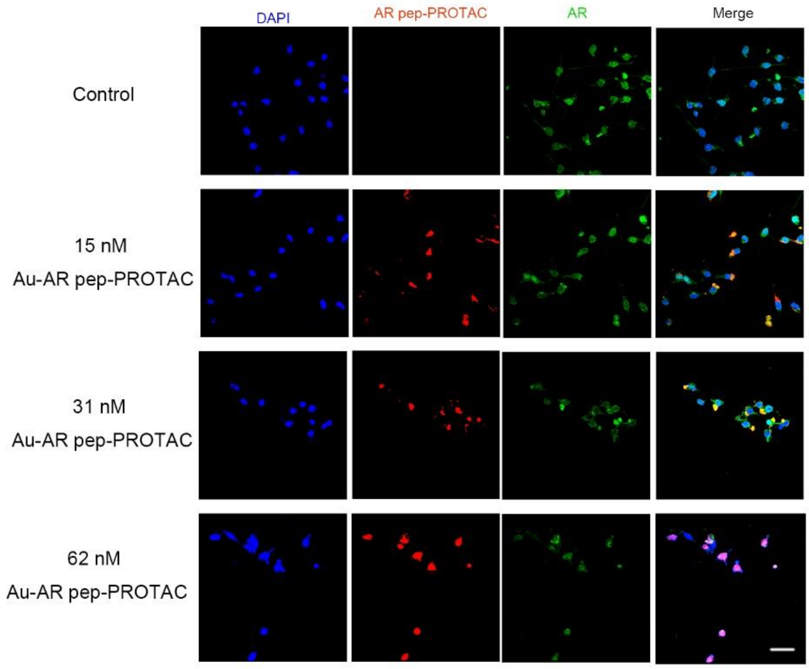

Figure S11. Co-location detection between AR pep-PROTAC and AR by confocal after 24h. The scale bar stands for 50 µm. DAPI was used for staining of the nucleus; Cy 5.5 (red) was used for AR pep-PROTAC label; FITC (green) was used for AR detection; Orange stands for co-location of AR pep-PROTAC and AR; purple stands for co-location of AR pep-PROTAC, AR and DAPI.

Figure S12

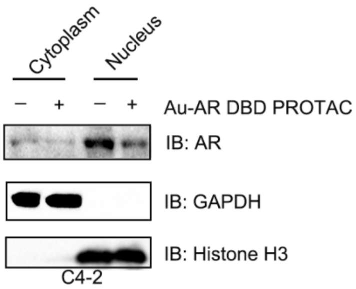

Figure S12. AR pep-PROTAC induced AR degradation in both of cytoplasm and nucleus.

Figure S13

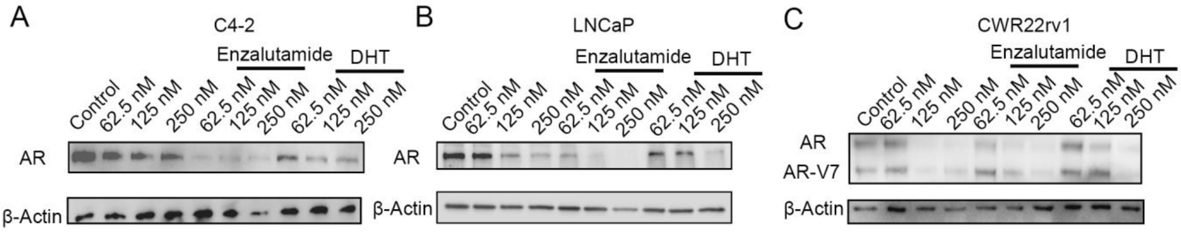

Figure S13. IB analysis of AR and AR-V7 in C4-2 (A), LNCaP (B), and CWr22rv1 (C) after Au-AR pep-PROTAC treatment in the presence of Enzalutamide or DHT.

Figure S14

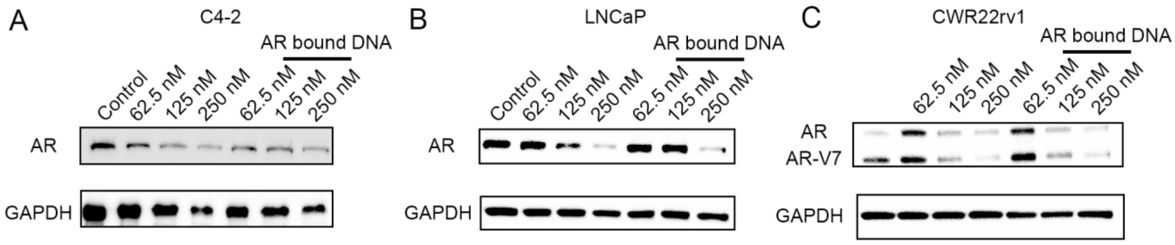

Figure S14. IB analysis of AR and AR-V7 in C4-2 (A), LNCaP (B), and CWr22rv1 (C) after Au-AR pep-PROTAC treatment in the presence of AR bound DNA. The double strands AR bound DNA was purchased from double strands and transfected with PEI.

Figure S15

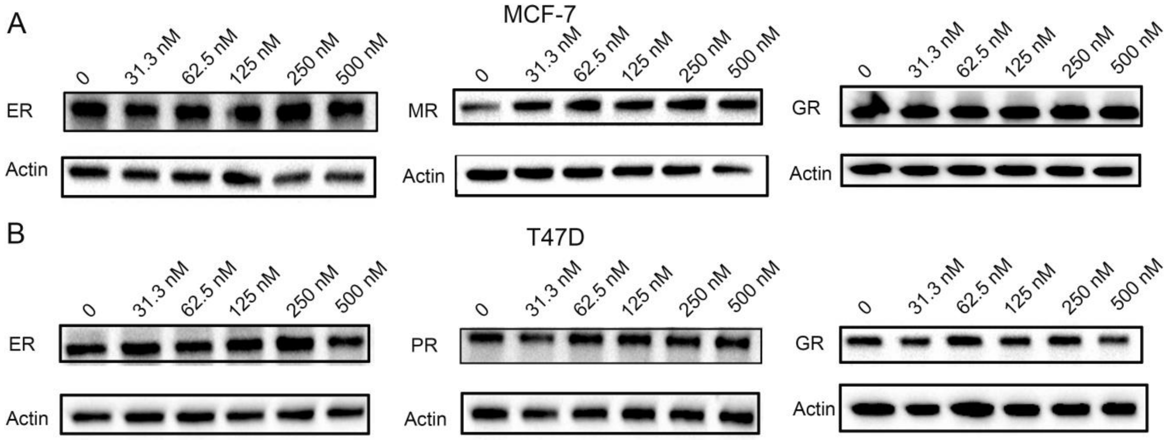

Figure S15. Au-AR pep-PROTAC showed no effect on ER, MR, GR, or PR protein levels. (A) Au-AR pep-PROTAC shows no ability to induce degradation of ER, MR, or GR in MCF-7 cells. **(B)** Au-AR pep-PROTAC shows no ability to induce degradation of ER, PR, or GR in T47D cells.

Figure S16

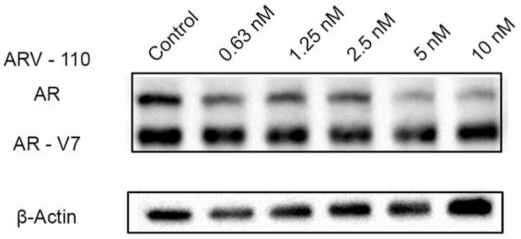

Figure S16. ARV-110 did not affect AR-V7 and only reduce full-length AR degradation. Immunoblotting (IB) analysis of AR and AR-V7 after ARV-110 treatment.

Figure S17

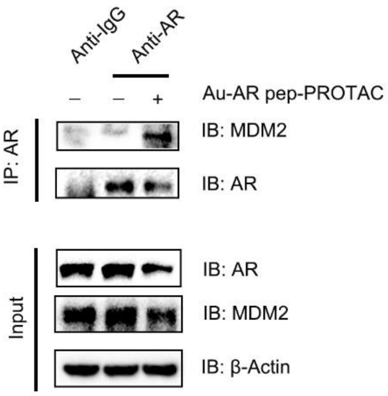

Figure S17. IB analysis of WCLs and anti-AR immunoprecipitate (IP) from

CWR22rv1 cells with or without AR pep-PROTAC drug treatment.

Figure S18

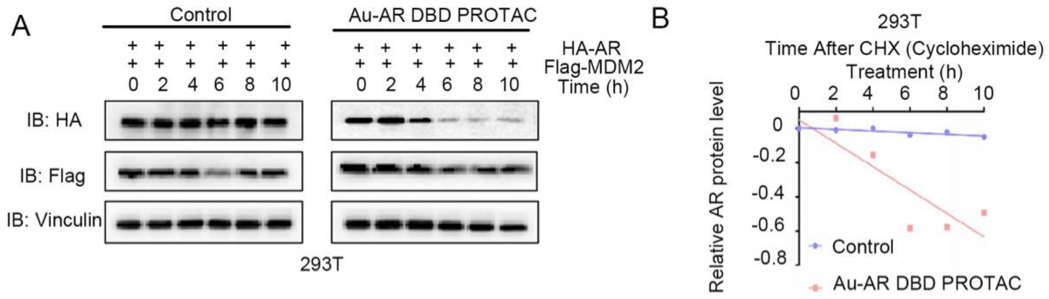

Figure S18. AR pep-PROTAC induces endogenous AR degradation in the presence of MDM2. (A) Immunoblotting analysis of whole-cell lysis (WCL) derived from 293T cells transfected with indicated plasmids (HA-tagged AR, Flag-tagged MDM2) in the presence of Control or 250nM AR-DBD PROTAC drug, treated with Cycloheximide (CHX) for indicated hours. (B) Quantified data of AR intensity normalized to Vinculin.

Figure S19

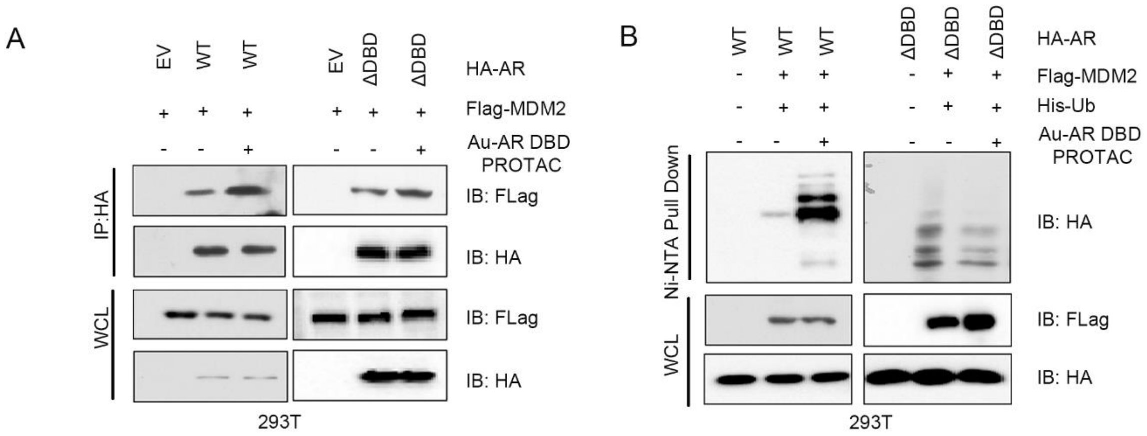

Figure S19. Au-AR pep-PROTAC drug induces AR degradation in a DBD-dependent way. (A) IB analysis of WCL and HA–immunoprecipitation (IP) derived from 293T cells transfected with indicated plasmids (HA-tagged AR, Flag-tagged MDM2) in the presence of Au-AR pep-PROTAC drug. **(B)** IB analysis of WCL and Ni–NTA pull-down derived from 293T cells transfected with indicated plasmids (HA-tagged AR, Flag-tagged MDM2, His-tagged ubiquitin) in the presence of Au-AR pep-

PROTAC drug.

Figure S20

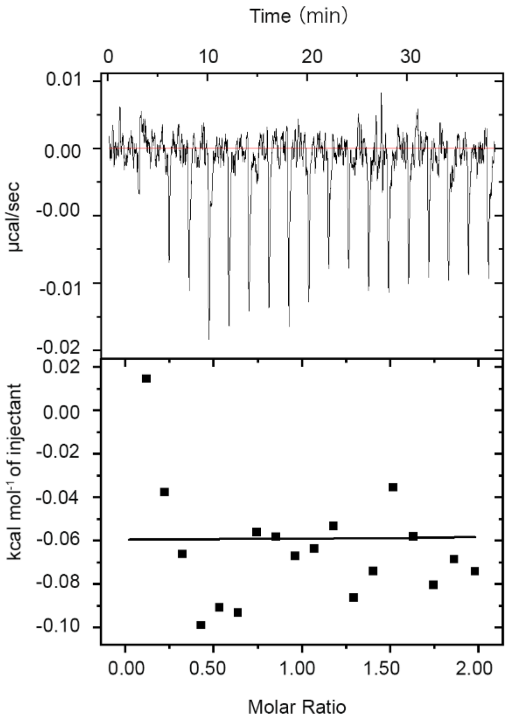

Figure S20. Binding affinity between AR pep-PROTAC and AR-ΔDBD measured by ITC.

Figure S21

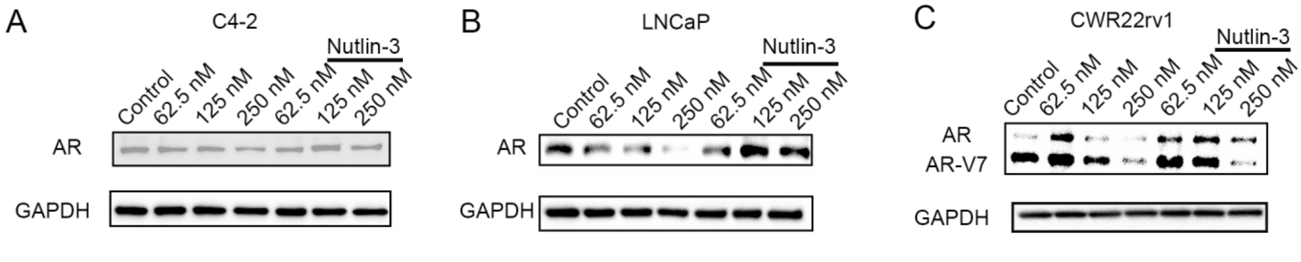

Figure S21. Nutlin-3 blocked Au-AR pep-PROTAC function.

Figure S22

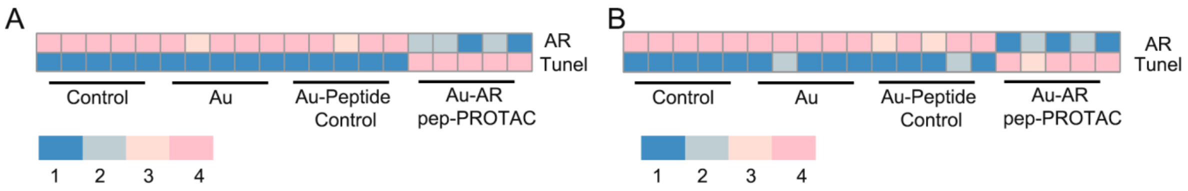

Figure S22. The correlation between depletion of AR proteins and apoptosis in tumor tissues from xenograft mice model after drug treatment.

Figure S23

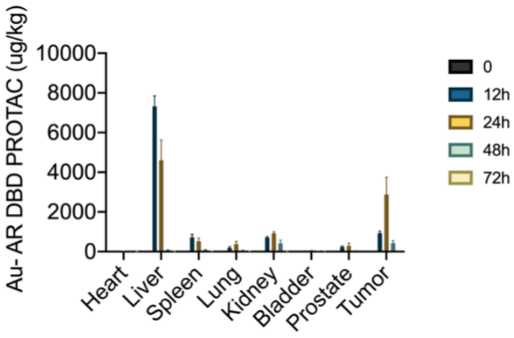

Figure S23. Pharmacokinetics and biodistribution analysis of Au-AR pep-PROTAC *in vivo* by ICP -MS.

Figure S24

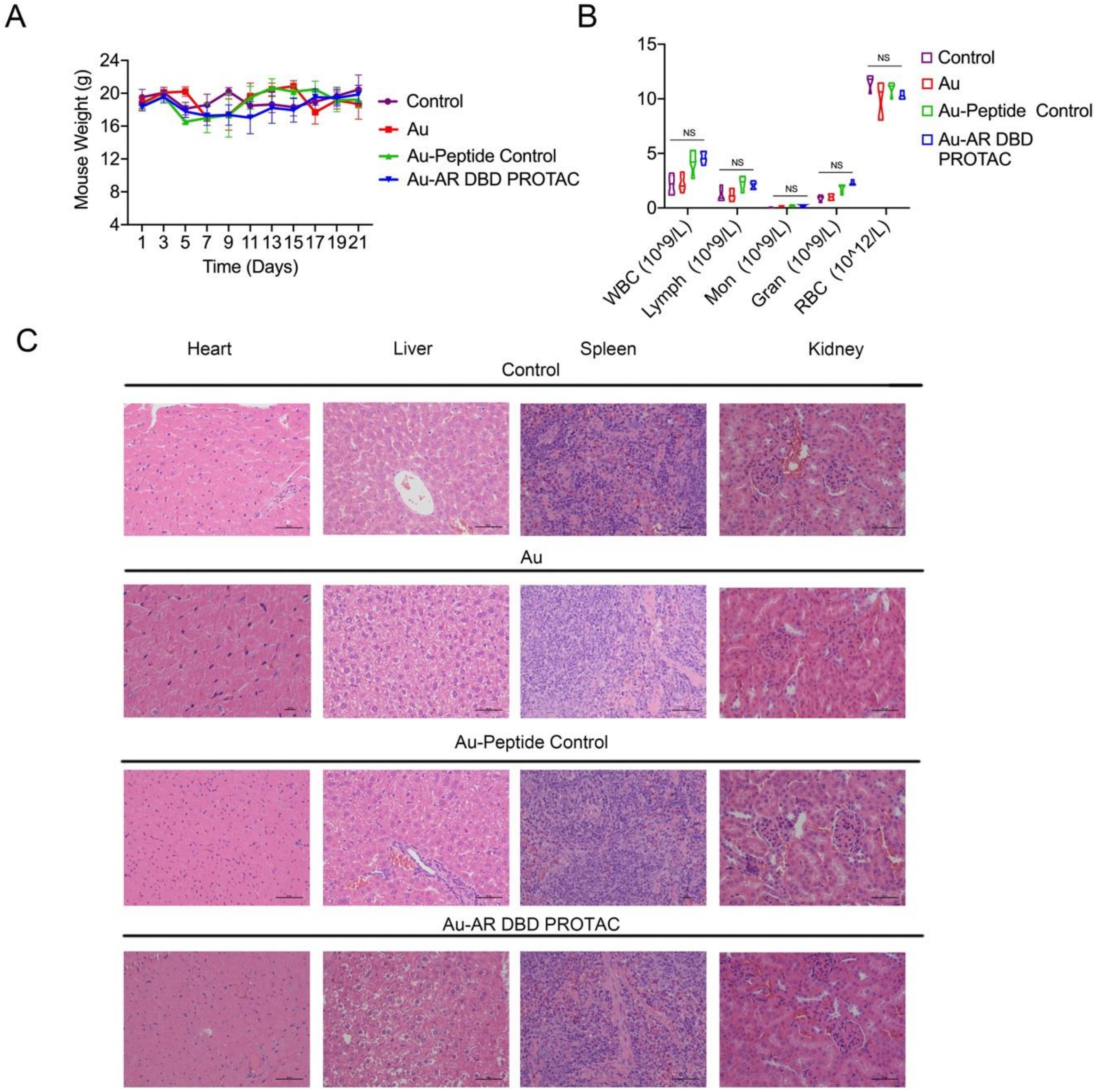

Figure S24. Safety evaluation of Au-AR pep-PROTAC *in vivo*. (A-B) Animal body weights **(A)** and counts of different types of blood cells **(B)** were detected after 21-day treatment of different drugs. WBC, white blood cell; Lymph, lymphocyte; Mon, monocyte; Gran, granulocytes; RBC, red blood cell. **(C)** Representative H&E staining photograph of heart, liver, spleen, and kidney sections from mice after the 21-day treatment of different drugs. Scale bar, 50 µm.

Figure S25

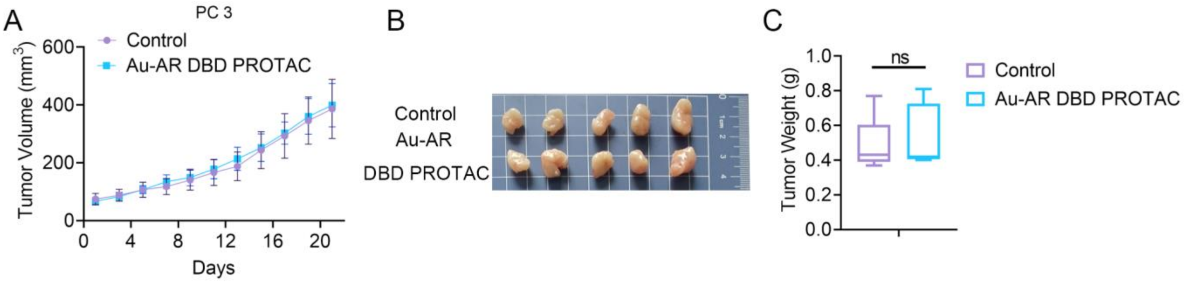

Figure S25. The Au-AR pep-PROTAC Drug could not inhibit negative prostate tumor growth *in vivo*. (**A)** Tumor growth curves of PC3 xenografts in nude mice treated as indicated (n=5 per group). **(B)** Photos of PC3 tumors excised at the end of the experiment after different drug treatments. **(C)** Average weight of tumors excised from each group of mice at the end of drug treatment. The data are presented as the mean ± SD values (n=5). Statistical analysis was performed using the nonparametric Kruskal-Wallis test; ns, not statistically significant.

Figure S26

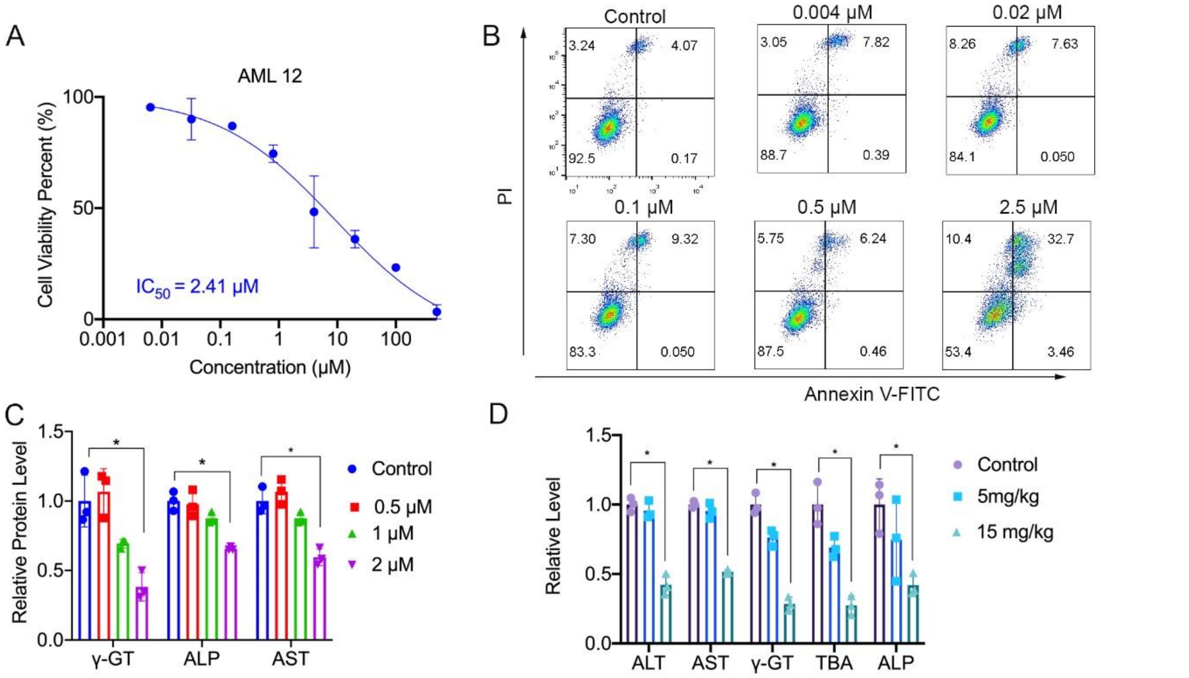

Figure S26. Toxicity evaluation of Au-AR pep-PROTAC on mice liver cells. (A)

Cell viability assay of AML 12 cells after 48 h of treatment with varying concentrations of the Au-AR pep-PROTAC. **(B)** Apoptosis detection of AML 12 cells after 24 h of treatment with varying concentrations of the Au-AR pep-PROTAC. **(C)** ELISA detection of γ-GT, ALP, and AST in AML 12 cell lysate after 24h treatment of Au-AR pep-PROTAC. **(D)** ELISA detection of γ-GT, ALP, AST, ALT, and TBA in mice serum after 24h injection of Au-AR pep-PROTAC.
